# Multi-omics to evaluate the protective mechanisms during *Akkermansia muciniphila* treatment of *Candida albicans* colonization and subsequent infection

**DOI:** 10.71150/jm.2502007

**Published:** 2025-08-31

**Authors:** Qiulin Luo, Huan Zhang, Youming Pu, Yingpu Wei, Jiangkun Yu, Xiaoshen Wang, Qin Cai, Ying Hu, Wenli Yuan

**Affiliations:** 1 Department of Clinical Laboratory, The Affiliated Hospital of Yunnan University (The Second People's Hospital of Yunnan Province), Kunming, Yunnan 650021, P. R. China; 2 Department of Clinical Laboratory, The Second Affiliated Hospital of Kunming Medical University, Kunming, Yunnan 650101, P. R. China; 3 Department of Clinical Laboratory, People's Hospital of Chuxiong city, Chuxiong, Yunnan 675000, P. R. China; 4 State Key Laboratory for Conservation and Utilization of Bio-Resources in Yunnan, School of Life Sciences, Yunnan University, Kunming, Yunnan 650500, P. R. China

**Keywords:** *Candida albicans*, gastrointestinal colonization, translocation infection, *Akkermansia muciniphila*, multi-omics analysis

## Abstract

*Akkermansia muciniphila* (AKK, *A. muciniphila*) fortifies the intestinal barrier, inhibits the colonization of pathogenic bacteria, and protects the host’s health. Nevertheless, the existing literature offers inadequate evidence to ascertain whether *A. muciniphila* can effectively treat *Candida albicans* (*C. albicans*) infections in vitro, and the underlying mechanisms remain ambiguous. This study, animal models were established through gavage with clinical isolates of *C. albicans* to induce gastrointestinal tract colonization and subsequent translocation infection. The models were subsequently administered *A. muciniphila*. We examined the analysis of 16S rRNA gene sequencing, metabolomics of colonic contents, and transcriptomics of colonic tissue. The intestinal barrier, inflammatory responses, and immune cell infiltration are analyzed. This study revealed that *A. muciniphila* markedly mitigated *C. albicans* translocation infection and modified the intestinal microbial community structure and metabolic attributes in model mice. After administering *A. muciniphila* to the translocation infection group, there was a notable increase in the prevalence of bacteria that produce short-chain fatty acids, including *Eubacterium_F*. Moreover, there was a significant increase in the levels of specific pathogens, including *Faecalibaculum*, *Turicibacter*, and *Turicimonas*. The study demonstrated that *A. muciniphila* treatment can improve the composition of intestinal microbiota and metabolites, augment the tight junctions of colonic tissue and diminish systemic inflammatory response. This presents an innovative therapeutic approach for the potential treatment of intestinal *C. albicans* infection using *A. muciniphila*.

## Introduction

*Candida albicans* (*C. albicans*) is a common fungal symbiont found on multiple surface barriers, such as the skin, oral cavity, gastrointestinal tract, and genital tract (Mirhakkak et al., 2020; Tan et al., 2019). *C. albicans* is regarded as an opportunistic pathogen (Witchley et al., 2021). In healthy individuals devoid of underlying medical conditions, *C. albicans* generally persists at its commensal locations without inducing symptomatic disease or requiring antifungal treatment (Swidergall et al., 2019). However, under particular circumstances—such as extended antibiotic administration, immunosuppressive treatment, hormonal fluctuations, high-sugar diets, or impaired mucosal barriers—it may evolve into a pathogenic state. This transition may lead to mucosal infections or invasive bloodstream infections, which can be fatal, especially in individuals with compromised immune systems or disrupted microbial equilibrium (Lass-Flörl and Steixner, 2023; Lass-Flörl et al., 2024). A recent study (Mesquida et al., 2023) utilizing species-specific microsatellite markers compared *C. albicans* genotypes from rectal swabs with previously collected and analyzed blood samples. This study identified the gastrointestinal tract as a potential reservoir for *Candida* spp. capable that can induce candidemia.

*Akkermansia muciniphila* (AKK, *A. muciniphila*) is a genus in the family Akkermansiaceae, which is part of the phylum Verrucomicrobia. *A. muciniphila* is a Gram-negative, strictly anaerobic bacterium typically present in vertebrates. It predominantly inhabits the mucus layer of the colonic and cecal mucosa, constituting 1% to 4% of the overall intestinal microbiota (Derrien et al., 2004). This bacterium can decompose host mucin in the intestinal tract, liberating amino acids and monosaccharides, producing short-chain fatty acids (SCFAs), and supplying nutrients for commensal intestinal flora (Ottman et al., 2017a; van der Lugt et al., 2019). *A. muciniphila* reduces mucin levels while simultaneously stimulating goblet cells to enhance mucin production, resulting in a thicker mucus layer (Derrien et al., 2016). This dual function improves epithelial integrity and diminishes the translocation of lipopolysaccharide (LPS) into systemic circulation. A recent study (Zhang et al., 2023) indicated that extracellular vesicles from *A. muciniphila* ATCC-BAA835 improved intestinal epithelial adhesion and decreased intestinal permeability in Caco-2 cells and an obesity mouse model. Previous studies (Hänninen et al., 2017; Vergalito et al., 2024) have demonstrated that in a murine model of antibiotic-induced intestinal microbiota disruption, the administration of live *A. muciniphila* markedly enhanced the diversity, structural richness, and homogeneity of the intestinal microbiota. Additionally, *A. muciniphila* was demonstrated to promote intestinal mucin secretion, strengthen intercellular junctions within the intestinal epithelium, and improve intestinal barrier integrity. These effects impede colonization by specific pathogenic bacteria, thus protecting host health. *A. muciniphila* is considered a promising candidate for the development of next-generation probiotics due to its potential as a therapeutic agent for microbial-related diseases. Our previous study (Zhang et al., 2024) utilized a multi-omics approach to identify markers of microbial-host interactions during *C. albicans* colonization and subsequent infection. Nonetheless, it is uncertain whether *A. muciniphila* significantly impacts *C. albicans* gastrointestinal colonization and subsequent infection.

To investigate this, we developed models for *C. albicans* gastrointestinal colonization and translocation infection, as well as treatment models incorporating *A. muciniphila*. We employed a multi-omics approach to examine variations in the microbiome, metabolic alterations, and host gene expression. The integrity of the intestinal barrier, inflammatory responses, and immune cell infiltration were thoroughly examined in colonic tissue from animal models administered *A. muciniphila* during *C. albicans* colonization and subsequent systemic infection. This study’s findings offer new insights into the clinical management of *C. albicans* gastrointestinal colonization and translocation infections. They also expand the potential applications of *A. muciniphila* in the prevention and treatment of microbial-related diseases.

## Materials and Methods

### Animals studies

Animal studies were conducted utilizing SPF grade C57BL/6 female mice, aged 6–8 weeks (20–24 g), which were procured and housed at the Yunnan University Laboratory Animal Center. Rearing conditions: stable temperature (24 ± 2℃), stable humidity (50 ± 5%), consistent circadian rhythm alternation, with unrestricted access to food and water for the mice. After 5 days of acclimatization feeding, mice were randomly assigned to the following groups: control (Control), *C. albicans* colonization (Colonization), *C. albicans* translocation (Translocation), *A. muciniphila* treatment with *C. albicans* colonization (AKK_Colonization), and *A. muciniphila* treatment with *C. albicans* translocation (AKK_Translocation), with 6 mice in each group. Prepare *C. albicans* broth by inoculating *C. albicans* in Yeast Peptone Dextrose medium (YPD) containing 1% yeast extract, 2% peptone, and 2% dextrose, and incubate at 25°C for 48 h. Sterile saline was rinsed twice, and then diluted to a concentration of 1 × 10^8^ CFU/ml. Preparation of *A. muciniphila* bacterial liquid: The standard strain *A. muciniphila* ATCC BAA-835 was acquired from the Guangdong Provincial Microbial Strain Preservation Center. The acquired strain was inoculated into *A. muciniphila*-specific liquid medium for a 3-day culture expansion, subsequently washed twice with sterile 0.9% sodium chloride solution, and diluted to 5 × 10^8^ CFU/ml using sterile saline. The administration of antibiotics and immunosuppressants facilitated the creation of a *C. albicans* gastrointestinal colonization model. Antibiotic administration commenced on the sixth day of the experiment, with each group of mice receiving levofloxacin (3 mg/ml) via gavage daily (0.2 ml/10 g) until the experiment's conclusion. Additionally, cyclophosphamide (150 mg/kg) was administered intravenously in the tail every other day, totaling five injections. *C. albicans* gavage commenced on the 20th day of the experiment, utilizing a bacterial solution concentration of 1 × 10^8^ CFU/ml administered once daily for five consecutive days. Mice were euthanized on day 29, and blood, intestinal contents, intestinal wall tissue, and kidneys were harvested and preserved in liquid nitrogen or immersed in formalin as per the experimental protocol. The translocation group model was derived from the colonization group, with cyclophosphamide (150 mg/kg) administered via the tail vein beginning on day 25, at intervals of every other day, for a total of five administrations, followed by identical subsequent treatments. The AKK_Colonization group and the AKK_Translocation group received *A. muciniphila* (5 × 10^8^ CFU/ml) via gavage beginning on day 6, in addition to the Colonization and Translocation groups, respectively, until *C. albicans* administration ceased one day prior to gavage, which was conducted once daily. All remaining treatments were identical. Mice were euthanized, and the contents of the colon, along with kidney and colonic tissue, were collected. Quantitative culture and RT-PCR were employed to assess the *C. albicans* load in the colonic contents and kidneys. Furthermore, colonic tissue underwent fungal fluorescence staining. Figure S1 demonstrates notable *C. albicans* proliferation and a significant increase in 18S rRNA expression within the colonic contents, confirming the successful establishment of *C. albicans* gastrointestinal colonization animal models. The colonic contents demonstrated a marked enhancement in *C. albicans* proliferation and 18S rRNA expression, while fluorescent staining of the colonic mucosal tissue revealed fungal spores, thereby validating the successful creation of an animal model for *C. albicans* gastrointestinal colonization. Moreover, the successful creation of a *C. albicans* translocation infection model was validated by the proliferation of *C. albicans* in renal tissue and notable expression of 18S.

### Compositional profiling of gut microbial community diversity

After the mice were executed, intestinal contents were rapidly frozen in liquid nitrogen and sent to Personalbio Technology for processing and Illumina high-throughput sequencing. Sequences were characterized utilizing the DADA2 method and assessed for Alpha and Beta diversity, in addition to profiling the bacterial community composition.

### Identification of Short-Chain Fatty Acids (SCFAs)

Mouse intestinal contents were collected, immediately frozen in liquid nitrogen, and transported to Personalbio Technology for processing and detection of short-chain fatty acids (SCFA) by GC-MS systems.

### Ultraperformance liquid chromatography-tandem mass spectrometry assay for nontargeted metabolomics

Mouse intestinal contents were collected, snap-frozen in liquid nitrogen and sent to Personalbio Technology for the processing and detection of metabolites using ultra-high-performance liquid chromatography-tandem mass spectrometry (UHPLC-MS/MS). Metabolites exhibiting substantial differences (VIP ≥ 1, *P* < 0.05) were identified through Variable Importance in Projection (VIP) and t-tests. Metabolic pathways were annotated using the Kyoto Encyclopedia of Genes and Genomes (KEGG) database (https://www.kegg.jp/kegg/pathway.html).

### Transcriptomic analysis of colonic tissue

Mice were executed, colonic tissue were harvested, rapidly frozen in liquid nitrogen, and examined by Personalbio Technology. Differential gene expression analysis was performed utilizing DESeq2, identifying differentially expressed genes (DEGs) based on |log₂FC| ≥ 1 and *P* < 0.05. KEGG pathway enrichment analysis was conducted on differentially expressed genes (DEGs).

### Reverse-transcription quantitative polymerase chain reaction (RT-PCR)

Total RNA extraction from intestinal wall tissue and contents was conducted utilizing the Axyprep Total RNA Small Volume Preparation Kit (Thermo Fisher Scientific, USA), following the reagent's instructions for the experiments. Reverse transcription of RNA was conducted using the RevertAid First Strand cDNA Synthesis Kit (Thermo Fisher Scientific, USA). RT-PCR was conducted utilizing the ABI-7500 (Applied Biosystems, USA).The total volume of RT-PCR was 20 μl, with the amplification conditions specified as follows: Amplification at 95°C for 3 min, succeeded by amplification at 95°C for 10 s and 60°C for 30 s over 40 cycles; the melting curves were generated under these cycling conditions: 95°C for 10 s and 65°C for 10 s, with a 0% increment per cycle. The melting curve was executed under the following cycling parameters: 95°C for 10 s and 65°C for 10 s, with a temperature increment of 0.5°C per cycle until reaching 95°C. Each sample was replicated thrice. The comparative expression of differential genes between groups was determined using the 2^-ΔΔCt^ method. The primers used for RT-PCR experiments are detailed in Table S1.

### ELISA

The concentrations of cytokines IL-6 and IL-10, along with occludin levels in colonic tissue, were quantified using ELISA kits (CusaBio, China; Ruixin Biotechnology, China) following the manufacturers’ protocols.

### Hematoxylin and eosin (HE) and immunohistochemical staining

Sections of paraffin-embedded fixed tissues were stained for HE and immunohistochemistry (CD68 [1:2000], CD19 [1:2000], CD56 [1:2000], and MPO [Original concentration]). Immunohistochemical results were observed by fluorescence microscope (Olympus).CD68, CD19, CD56, and MPO positive staining were all in the cytoplasm. Judging criteria: (1) According to the intensity of cell color development in the section: 0 score, no cell color development; 1 point, the cells are light yellow; 2 points, the cells are brownish yellow; 3 points, the cells are brown; (2) Score according to the proportion of positive cells: 0 points, positive cells < 5%; 1 score, positive cells < 25%; 2 points, positive cells 25–50%; 3 points, positive cells > 50%, and the sum of the two as the final judgment result.

### Statistical analysis

Data analysis and visualization were conducted utilizing SPSS 26.0 and GraphPad Prism 9.5.1. Statistical significance was assessed using one-way ANOVA, t-tests, or non-parametric Wilcoxon rank-sum tests.

## Result

### Treatment with *A. muciniphila* alters the diversity and composition of gut microbiota in *C. albicans* colonization and translocation infection

**Fig. 1.**
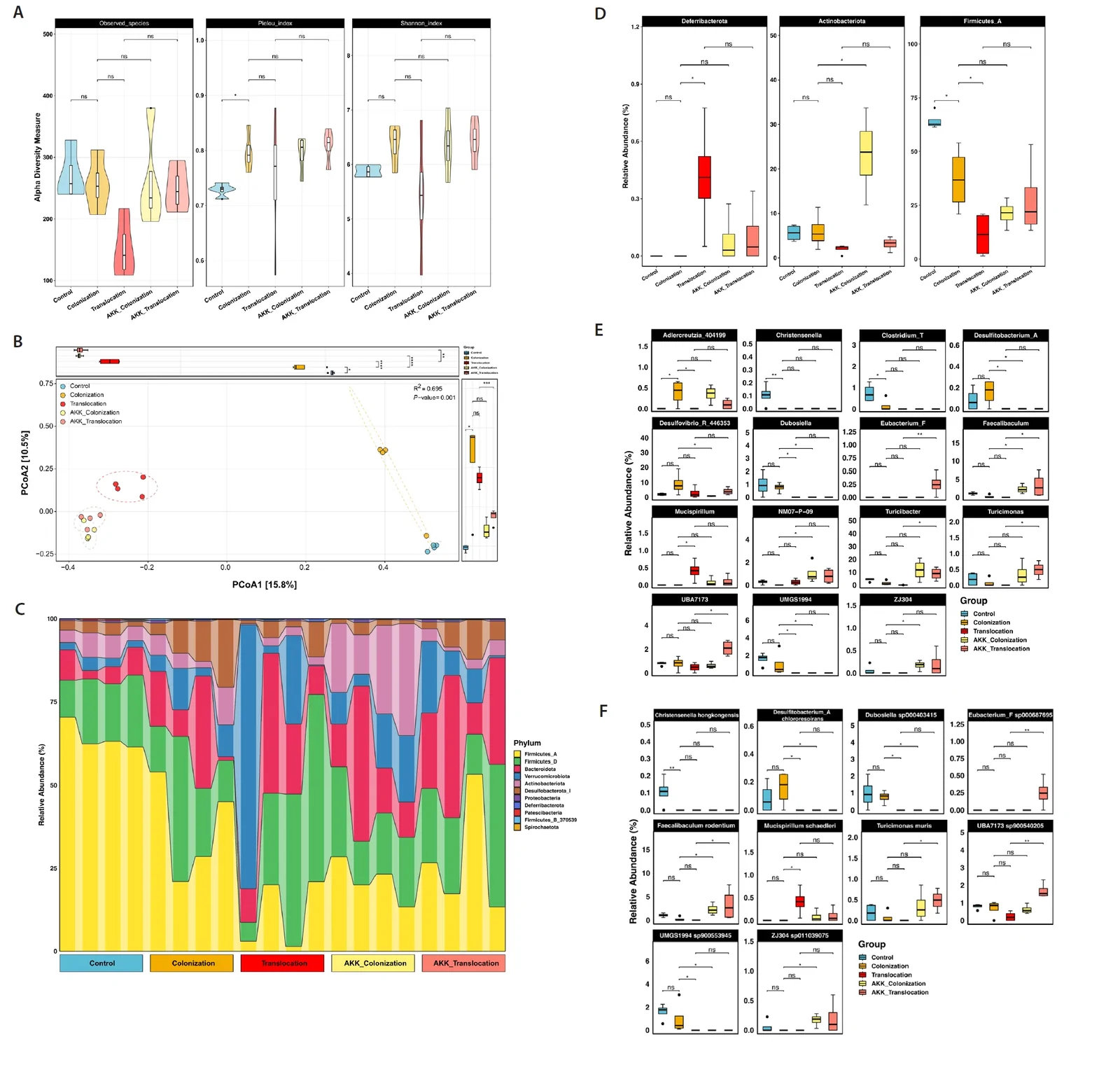
16S rRNA sequencing of colonic contents in model mice. (A) α-diversity is quantified by Observed Species, the Pielou index, and the Shannon index. (B) β diversity was illustrated through principal coordinate analysis (PCoA). (C) The relative abundance of predominant bacteria in the intestinal contents of mice across each experimental group is illustrated at the phylum level. (D) Variations in the relative abundance of predominant intestinal bacteria at the phylum level among mice from each experimental group. (E–F) Differences in relative abundance of mouse intestinal bacteria at the genus level and species level in each experimental group (^*^P < 0.05, ^**^P < 0.01).

To assess the impact of *A. muciniphila* treatment on *C. albicans* gastrointestinal colonization and translocation infection regarding the diversity and composition of the bacterial community, 16S rRNA gene amplicon sequencing was performed on colonic contents from the five groups. Alpha diversity was assessed using the Observed Species, Pielou, and Shannon indices. No significant difference (*P* > 0.05) was observed in alpha diversity (Fig. 1A). Principal coordinate analysis (PCoA) utilizing Jaccard distance demonstrated that *A. muciniphila* significantly influenced the gut microbial community structure during *C. albicans* colonization and translocation infection. Of greater concern was the disappearance of significant differences in microbial community structure caused by *C. albicans* colonization and translocation infections with the intervention of *A. muciniphila* (PERMANOVA: R2 = 0.695, *P* = 0.001), as depicted in Fig. 1B.

Differences in the relative abundance of microorganisms among the five groups were analyzed. At the phylum level, the relative abundance of Actinobacteria was significantly elevated (*P* < 0.05) in the AKK_*C. albicans* colonization group compared to the *C. albicans* colonization group, as depicted in Fig. 1D. At the genus and species level, AKK_*C. albicans* colonization group showed a significant increase in *Faecalibaculum*, *NM07−P−09*, *Faecalibaculum rodentium*, *ZJ304* and *ZJ304 sp011039075*, conversely *Desulfitobacterium_A*, *Desulfovibrio_R_446353*, *Desulfitobacterium_A Chlororespirans*, *Dubosiella sp000403415*, *UMGS1994 sp900553945*, and *Dubosiella* were significantly decreased compared to *C. albicans* colonization group. In addition, both *Eubacterium_F*, *Faecalibaculum*, *Turicibacter*, *Turicimonas*, *Eubacterium_F* sp000687695, *Faecalibaculum rodentium*, *Turicimonas* muris, *UBA7173 sp900540205*, and *UBA7173* relative abundance were significantly increase (*P* < 0.05) in the AKK_*C. albicans* translocation group compared to the *C. albicans* translocation group (Fig. 1E and 1F).

### *A. muciniphila* treatment alters the metabolomics of *C. albicans* colonization and translocation infection

**Fig. 2.**
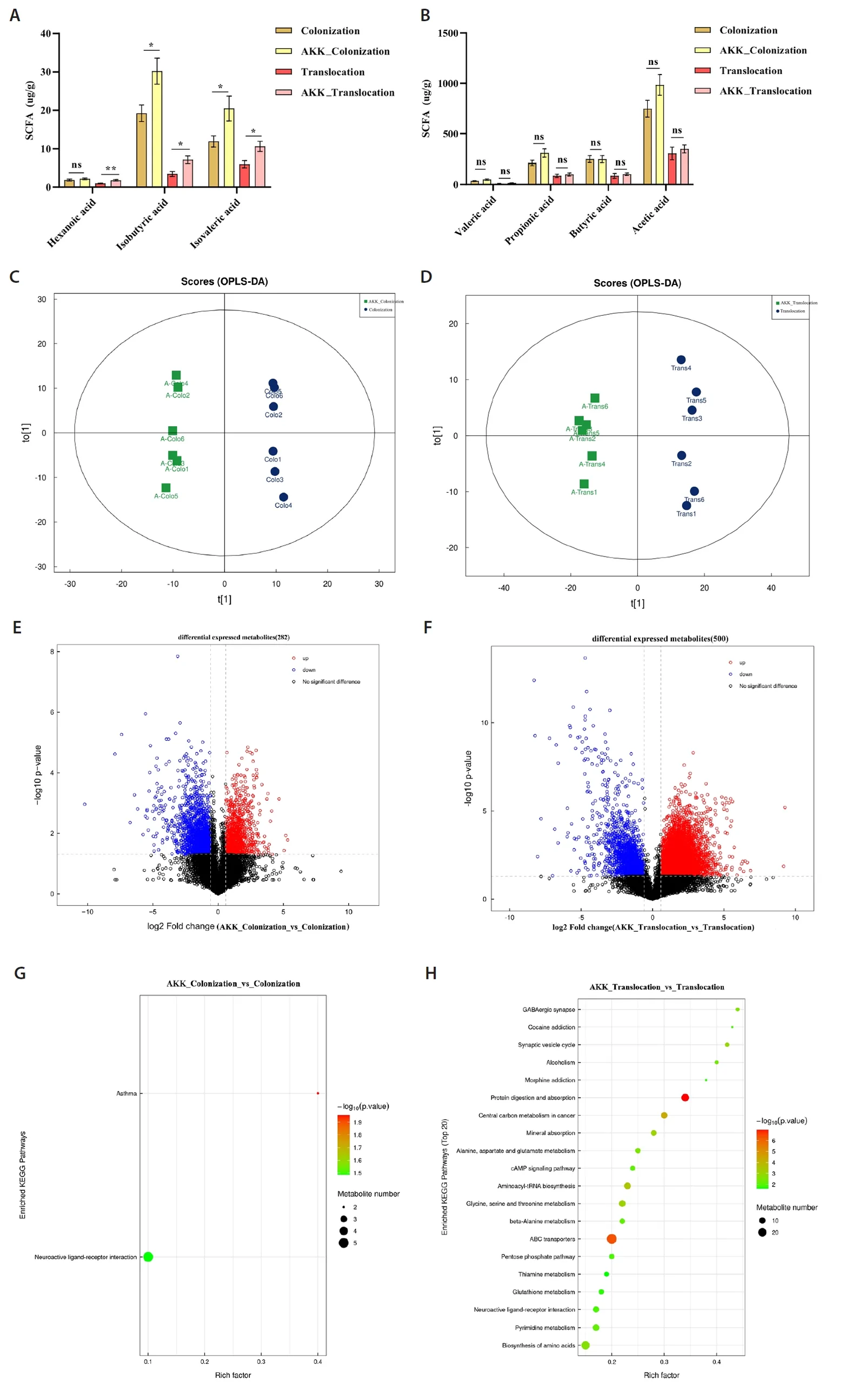
Metabolomic analysis of intestinal contents of experimental mice. (A–B) Statistical analysis of SCFAs levels in intestinal contents (^*^P < 0.05). (C–D) Comparison of the differences in intestinal metabolic profiles between AKK intervention mice and non-intervention mice by OPLS-DA analysis. (E–F) Volcano plots illustrating metabolite disparities between mice in the AKK intervention group and those in the non-intervention group. Red circles denote metabolites that are significantly up-regulated (*P* < 0.05, FC ≥ 2); blue circles indicate metabolites that are significantly down-regulated (*P* < 0.05, FC ≤ 0.5); black circles represent metabolites with no significant difference. (G–H) KEGG enrichment analysis was conducted to examine the primary enrichment pathways of differential metabolites between mice subjected to AKK intervention and those without intervention.

Short-chain fatty acids (SCFAs) are essential metabolites primarily generated by the gut microbiota. Treatment with *A. muciniphila* in the *C. albicans* colonization and translocation infection model led to a marked elevation of isobutyric and isovaleric acid concentrations in the intestinal contents of mice, as illustrated in Fig. 2A.

This study utilized UHPLC-MS/MS for non-targeted metabolomic analysis of colonic contents samples from models to ascertain whether *A. muciniphila* treatment influences *C. albicans* colonization and translocated infection, thereby inducing metabolic alterations in feces. We additionally assessed the disparities in metabolic profiles between the *A. muciniphila* intervention and non-intervention groups using two-dimensional Principal Component Analysis (PCA) and Orthogonal Partial Least Squares Discriminant Analysis (OPLS-DA) analyses (Fig. 2C and 2D). This experiment identified 2871 metabolites, with 282 differential metabolites observed in the *C. albicans* colonization group and the AKK_*C. albicans* colonization group. Among these, 86 metabolites were up-regulated and 196 were down-regulated (Fig. 2E). A total of 500 differential metabolites were identified in the *C. albicans* translocation infection group and the AKK_*C. albicans* translocation infection group, with 348 metabolites exhibiting up-regulation and 152 metabolites demonstrating down-regulation, as illustrated in Fig. 2F. This study enumerated the top ten differential metabolites between the two groups, as presented in Tables 1 and 2, respectively. This study conducted KEGG enrichment analysis of the differential metabolites and identified metabolic pathways with significant discrepancies between groups. The primary pathways enriched in the AKK_*C. albicans* colonization group, relative to the *C. albicans* colonization group, were the Neuroactive ligand-receptor interaction and Asthma pathways, as depicted in Fig. 2G, with the expression of the pertinent differential metabolites up-regulated across all pathways. The primary substances included Prostaglandin I2, Leukotriene D4, gamma-aminobutyric acid, Histamine, D-erythro-sphinganine-1-phosphate, and Leukotriene D4. The pathways enriched in the AKK_*C. albicans* translocation infection group, relative to the *C. albicans* translocation infection group, primarily included Protein digestion and absorption, ABC transporters, Central carbon metabolism in cancer, Aminoacyl-tRNA biosynthesis, Glycine, serine and threonine metabolism, Alanine, aspartate and glutamate metabolism, and the AKK_*C. albicans* translocation infection group. The metabolism of glutamate, beta-alanine, and neuroactive ligand-receptor interactions (Fig. 2H) exhibited an upregulation in the expression of associated differential metabolites.

### Transcriptomic profiling of the colonic tissue

**Fig. 3.**
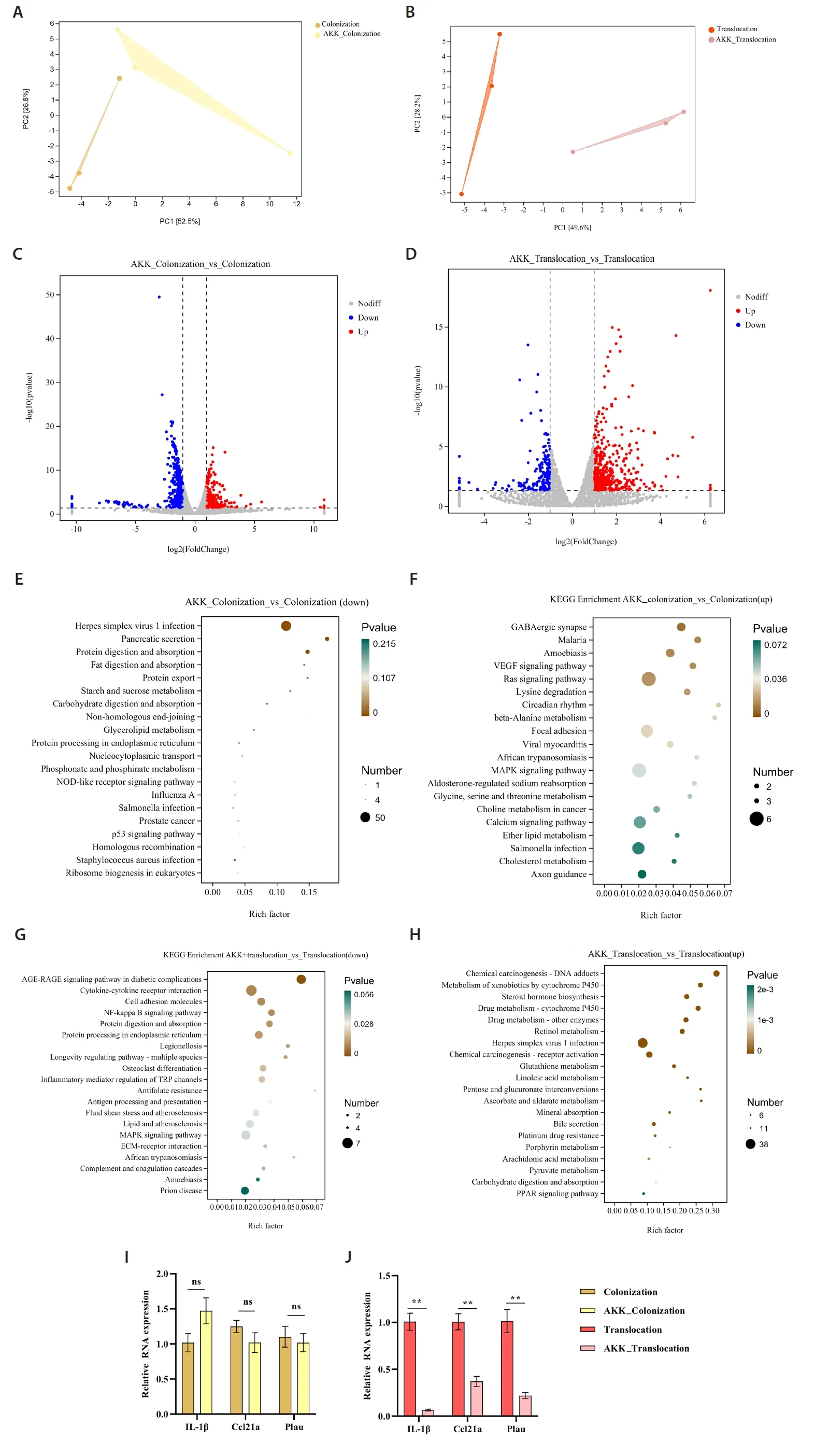
Transcriptomic analysis of murine colonic tissues. (A–B) Principal component analysis comparing AKK intervention and non-intervention groups. (C–D) Volcano plots of differential gene expression between AKK intervention and non-intervention groups. Red dots denote genes that are significantly up-regulated (*P* < 0.05, FC ≥ 2); blue dots indicate genes that are significantly down-regulated (*P* < 0.05, FC ≤ 0.5); gray dots represent genes with no significant difference. (E–H) Bubble plots illustrating KEGG pathways primarily enriched for differentially expressed genes, encompassing both up-regulation and down-regulation. (I–J) The reliability of RNA-Seq concerning gene expression was validated through RT-PCR. The RNA-Seq technique exhibited a high degree of concordance with the RT-PCR method (^**^P < 0.01).

Principal Component Analysis (PCA) identified transcriptomic variations between the *A. muciniphila* treatment group and the *C. albicans* colonization and translocation infection groups. This study demonstrated that the samples were closely grouped, signifying high repeatability and minimal dispersion, suitable for further analysis (Fig. 3A and 3B). Figure 3C illustrates that a total of 711 genes were differentially expressed at a fold change (FC) ≥ 2 or ≤ 0.5 with a significance level of *P* < 0.05 in the *C. albicans* colonization group compared to the AKK_*C. albicans* colonization group. Among these, 432 genes were down-regulated and 279 genes were up-regulated. Figure 3D illustrates that a total of 637 genes exhibited differential expression between the *C. albicans* translocation infection group and the AKK_*C. albicans* translocation infection group. Genes 181 and 456 were down-regulated and up-regulated, respectively. Subsequent transcriptome data analysis encompassed Kyoto Encyclopedia of Genes and Genomes (KEGG) enrichment analyses. The twenty most significantly enriched KEGG pathways for up-regulation and down-regulation (*P* < 0.05) were chosen for presentation. In comparison to the *C. albicans* colonization group, the down-regulated genes were predominantly enriched in Herpes simplex virus 1 infection, pancreatic secretion, protein digestion and absorption, and fat digestion and absorption (Fig. 3E). Up-regulated genes in the AKK_*C. albicans* colonization group were predominantly enriched in GABAergic synapse, malaria, amoebiasis, VEGF signaling pathway, Ras signaling pathway, beta-alanine metabolism, and MAPK signaling pathway (Fig. 3F). The differentially expressed genes (DEGs) in the *C. albicans* translocation infection group, when compared to the AKK_*C. albicans* translocation infection group, the pathways predominantly enriched for down-regulated genes included the NF-κB signaling pathway, the AGE-RAGE signaling pathway in diabetic complications, and cytokine-cytokine receptor interaction (Fig. 3G). Exhibited significant enrichment in pathways related to chemical carcinogenesis - DNA adducts, metabolism of xenobiotics by cytochrome P450, and steroid hormone biosynthesis. Metabolism - alternative enzymatic pathways (Fig. 3H).

The study also evaluated the expression levels of the indicator genes IL-1β, Ccl21α, and Plau through real-time quantitative PCR (Fig. 3I and 3J). RT-PCR analysis demonstrated that compared to the *C. albicans* translocation infection group, the AKK_*C. albicans* translocation infection group exhibited significantly reduced expression levels of the inflammatory-related genes Plau and IL-1β, as well as the Ccl21a. Furthermore, these results demonstrate significant consistency between RNA-Seq and RT-PCR in gene expression quantification, validating the reliability of RNA-Seq for relative gene expression analysis.

### Multi-omics analysis of the microbe-metabolite-host axis in *A. muciniphila* treatment *C. albicans* colonized and subsequently translocated infected mice

**Fig. 4.**
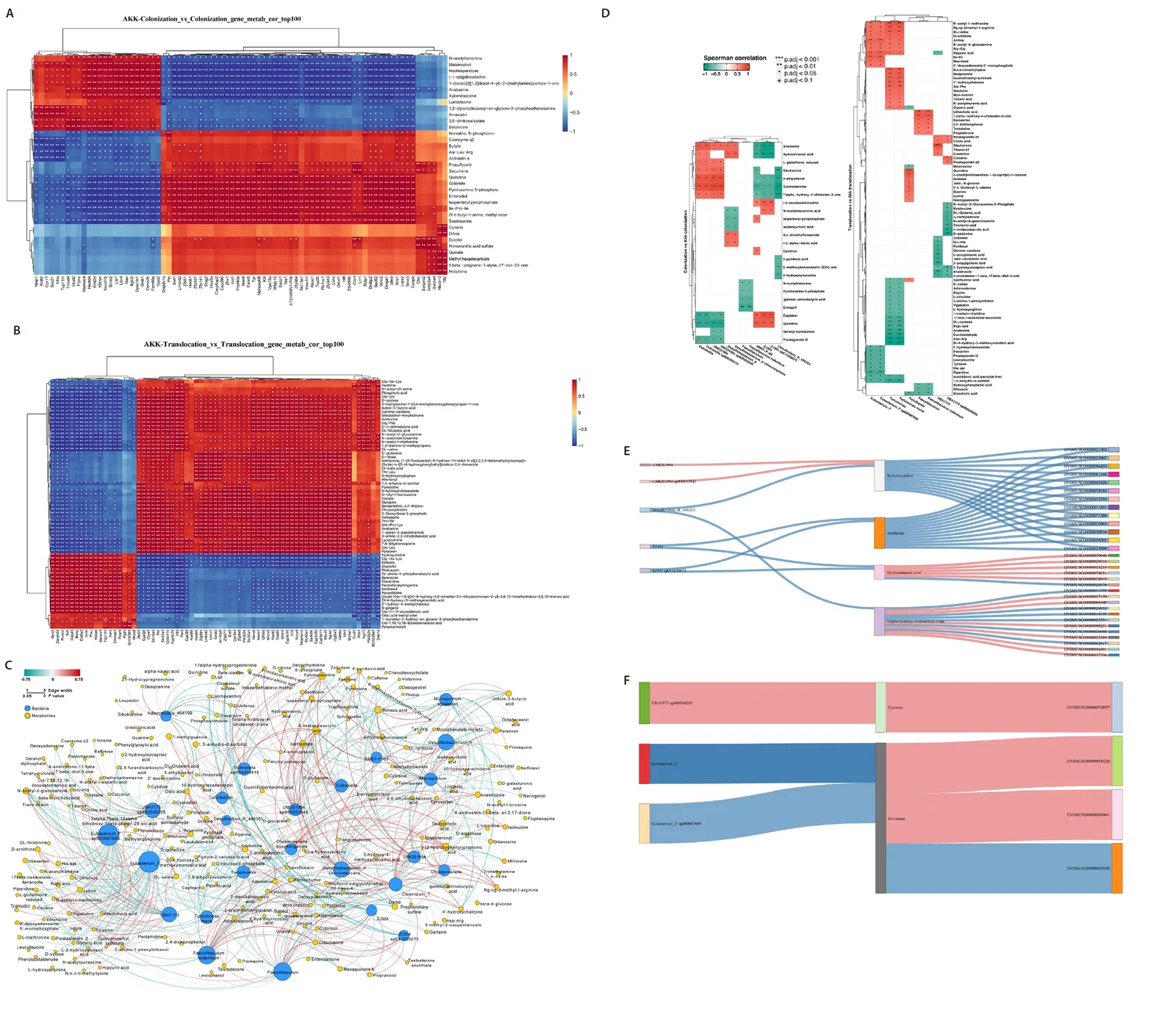
Multi-omics analysis of AKK intervention in *C. albicans* gastrointestinal tract colonization and translocation in infected mice. (A–B) Correlation analysis of the top 100 differential genes with differential metabolites in AKK intervention and non-intervention groups. (C–D) Correlation analysis of differential bacteria with differential metabolites in AKK intervention and non-intervention groups of mice. (E–F) A Spearman correlation analysis was conducted to examine the potential relationships among differential bacteria, differential metabolites, and differential genes between the AKK-intervened *C. albicans* colonized group and the *C. albicans* colonized group, as well as between the AKK-intervened *C. albicans* translocated infected group and the *C. albicans* translocated infected group, with pink indicating a positive correlation and blue indicating a negative correlation.

To evaluate the impact of *A. muciniphila* treatment on gut microbiota and host metabolism related to *C. albicans* colonization and translocation infections, we independently examined the effects of *A. muciniphila* treatment on *C. albicans* colonization and subsequent translocation infections during the colonization phase, analyzing the colonic contents of indicator bacterial genera and differential non-target metabolites from the samples. A correlation was established between the top 100 differential genes and differential metabolites in the *A. muciniphila* treatment and non-treatment groups, respectively (Fig. 4A and 4B). Additionally, the association between 12 distinct bacterial genera and the differential metabolites extracted from the colonic contents in the *C. albicans* colonization group and the AKK_*C. albicans* colonization group was examined. Nonetheless, *Desulfovibrio_R_446353* and *Faecalibaculum* exhibited a paradoxical negative correlation with all Differentially Abundant Metabolites (DAMs). There were both positive and negative correlations between certain DAMs (7.alpha.−hydroxy−4−cholesten−3−one, Xylometazoline, Hydrocinnamic acid, and Anabasine) and the identified bacterial genera. Conversely, *Desulfovibrio rodentium* exhibited a paradoxical negative association with all DAMs. We also examined the correlation between nine distinct bacterial genera and the differential metabolites extracted from the colonic contents in the AKK_*C. albicans* translocation infection group and the *C. albicans* translocation infection group (Fig. 4D). This experiment investigated whether *A. muciniphila* treatment could influence gene expression in *C. albicans*-colonized and translocated infected mice by modifying microbial ecology and bacterial metabolites. A Spearman correlation analysis was utilized to determine potential correlations among all differential transcripts, metabolites, and bacterial genera. The integration of *C. albicans* gastrointestinal colonization with the findings from the indicator bacterial genus, metabolomic, and transcriptomic variations in the AKK_*C. albicans* gastrointestinal colonization group was employed to examine the correlations among five bacteria, four metabolites, and 31 genes. Correlation analyses revealed intricate interactions between the microbiome and the host (Fig. 4E). A correlation analysis was performed between three differential bacterial genera, two distinct metabolites, and four genes, utilizing the combined data from the *C. albicans* translocation infection group and the AKK_*C. albicans* translocation infection group (Fig. 4F). The reconstruction of the co-occurrence network through Spearman correlation analysis identified one positive correlation and two negative correlations, highlighting the complex interaction between the microbiome and the host.

## Discussion

The colonization and invasion of intestinal *C. albicans* are typically considered prerequisites for disseminated *C. albicans* infection (Hu et al., 2022; Mesquida et al., 2024). The results from our animal studies and clinical trial confirm that gastrointestinal colonization by pathogens is a crucial precursor to the onset of translocation-associated infections (Yuan et al., 2022; Zhang et al., 2024).

*A. muciniphila* plays a crucial role in regulating gut microbiota and metabolites, thereby safeguarding the intestinal tract and preventing *C. albicans* infections. This study investigates the impact of microbial ecological alterations and bacterial metabolites on the host during *A. muciniphila* treatment of *C. albicans* colonization and translocation infections. Our findings indicated that this intervention led to the upregulation of *Eubacterium_F*, *Faecalibaculum*, *Turicibacter*, and *Turicimonas* within the modified microbial ecosystem, presumably enhancing immune status. *Eubacterium_F* is recognized for its ability to metabolize intestinal nutrients, thereby augmenting energy extraction from the intestines, and is involved in various pathways, including immune regulation and inflammation suppression (Cattaneo et al., 2017; Mukherjee et al., 2020). This study found significantly elevated concentrations of hexanoic acid, isobutyric acid, and isovaleric acid in the colonic contents of the *A. muciniphila*-treated *C. albicans* translocation infection group, along with an increase in occludin content in colonic tissue. Multiple studies (Gasaly et al., 2021) have shown that SCFAs enhance barrier functionality by upregulating TJ protein expression, leading to elevated transepithelial electrical resistance. *Eubacterium_F*, a prevalent short-chain fatty acid-producing bacterial species (Lu et al., 2022), may have contributed to the increased concentrations of hexanoic acid, isobutyric acid, and isovaleric acid noted in the *A. muciniphila*-treated translocation infection cohort. SCFAs are recognized for their ability to influence gut inflammation by enhancing intestinal integrity and modulating immune responses. Moreover, SCFAs have demonstrated the ability to enhance transepithelial resistance by increasing the expression of tight junction proteins, including claudin-1 and occludin (Wang et al., 2012).

This study evaluated the intestinal barrier integrity of each experimental group by analyzing the concentration of intestinal tight junction proteins, as these concentrations indicate the integrity of the intestinal mucosa at the protein level. The occludin concentration in the translocation infection group was significantly lower than that in the colonization group. Nevertheless, subsequent to *A. muciniphila* treatment, occludin levels in the translocation infection group exhibited a significant increase. This discovery indicates that *A. muciniphila* promotes the expression of tight junction proteins in colonic tissue, aiding in the repair of intestinal mucosal damage, in accordance with prior research (Bian et al., 2019). Previous study (Ottman et al., 2017b) have suggested that TLR-2-mediated augmentation of tight junctions in intestinal epithelial cells may represent a potential mechanism for the *A. muciniphila*-dependent enhancement of gut barrier function. Furthermore, inflammation was evaluated by analyzing the levels of IL-6 and IL-10 in colonic tissue, along with the expression of CD19, CD56, CD68, and MPO. The findings indicated that *A. muciniphila* intervention markedly diminished IL-6 levels in mice infected with *C. albicans* and exposed to translocation. Numerous studies indicate that SCFAs function as anti-inflammatory agents by suppressing the synthesis of pro-inflammatory cytokines (Xiao et al., 2024; Yang et al., 2020).

A notable disparity in CD68+ macrophages was detected between the *C. albicans* translocation infection cohort and the *A. muciniphila*-treated translocation infection cohort, indicating that *A. muciniphila* markedly suppresses the infiltration of CD68+ macrophages. Previous studies (Panova et al., 2021; Wang et al., 2022; Yu et al., 2021) have shown that factors such as TNF-α, STAT1/STAT3, IL-1β, IL-10, and MCP-1 (CCL-2) are closely associated with CD68 expression. Among these, TNF-α and IL-1β upregulate CD68 expression by activating multiple signaling pathways, while MCP-1 (CCL-2) promotes the migration of monocytes and their differentiation into macrophages, demonstrating a positive correlation with CD68 expression. Furthermore, oral gavage of *A. muciniphila* significantly reduces colonic expression of IL-1β and TNF-α while upregulating IL-10 in gp120 transgenic mice (Luo et al., 2025). In a TNBS-induced enteritis model, *A. muciniphila* alleviated intestinal inflammation by suppressing the expression of p-STAT3 and STAT3 (Jiang et al., 2025). In DSS-induced acute colitis mice, *A. muciniphila* administration decreased MCP-1 (CCL-2) levels (Qu et al., 2021). Plau enhances macrophage migration and promotes M1-type polarization, resulting in upregulated CD68 expression (Li et al., 2021). Furthermore, Plau modulates IL-6 levels through the NF-κB signaling pathway (Meng et al., 2024). Based on these findings, we hypothesize that *A. muciniphila* may modulate CD68 expression through regulation of these genes. Overall, the translocation infection cohort demonstrated greater intestinal damage and inflammation relative to the *A. muciniphila*-treated cohort. Nevertheless, *A. muciniphila* treatment ameliorated these symptoms, implying that *A. muciniphila* promotes intestinal tissue repair and augments intestinal flora activity, thereby alleviating intestinal damage induced by *C. albicans*. This finding was further validated in Fig. S2 of our study.

Transcriptomic and metabolomic analyses demonstrated that *A. muciniphila* treatment modified the expression of particular genes in the *C. albicans* translocation infection group. KEGG pathway analysis revealed *A. muciniphila* significantly suppressed NF-κB signaling pathway activity during treatment of *C. albicans* translocation infection. Concurrently, the downregulation of gene expression associated with cell adhesion molecules and the regulation of inflammatory mediators affecting TRP channels. Previous studies have demonstrated that *Akkermansia muciniphila* reduces the phosphorylation level of NF-κB p65, thereby inhibiting NF-κB pathway activation and mitigating inflammatory responses (Shi et al., 2022a, 2022b). The membrane protein AMUC_1100 of *Akkermansia muciniphila* significantly reduces the expression of pro-inflammatory cytokines IL-1β, TNF-α, and IL-6 by inhibiting the NF-κB signaling pathway (Wang et al., 2024). Nevertheless, the exact mechanisms of NF-κB regulation by *A. muciniphila* require further investigation. A previous study (Reunanen et al., 2015) indicated that *A. muciniphila* exhibits strong adhesion to intestinal cells, markedly enhancing the integrity of the intestinal epithelial monolayer. The attachment of probiotics to intestinal cells and their colonization of the gastrointestinal tract are crucial for providing beneficial effects to the host. Thus, we propose that *A. muciniphila* supplementation in individuals with *C. albicans* infection may improve gut integrity and promote human health. Additional research is necessary to explore the fundamental mechanisms. Concurrently, substantial changes in intestinal metabolism were noted among the groups. Prior studies have demonstrated a strong association between gut metabolites and various intestinal and extraintestinal disorders, such as inflammatory bowel disease, intestinal *C. albicans* infection, and irritable bowel syndrome (Hu et al., 2022). This research revealed significant disparities in intestinal metabolite profiles among the groups. KEGG data analysis identified 20 unique metabolic pathways, notably focusing on amino acid metabolism, encompassing the metabolism of serine, threonine, alanine, aspartate, and glutamate. Glutamine is essential for sustaining normal cellular metabolism, acting as a precursor in protein synthesis and aiding in cellular homeostasis. This study indicates that the downregulation of glutamine adversely affects the proliferation of *C. albicans*, underscoring its essential role in fungal biology. IL-1β has been shown to induce the production of additional cytokines and inflammatory mediators via autocrine or paracrine pathways (Seo et al., 2015; Xia et al., 2021). This activation subsequently enhances complement activity and exacerbates immune-mediated tissue damage. IL-1β promotes IL-6 secretion by activating the IL-6 promoter through modulation of the NF-κB signaling pathway (Liu et al., 2015). Moreover, IL-1β promotes the expression of adhesion molecules on vascular endothelium, facilitating the infiltration of inflammatory cells, into inflamed colonic tissue, resulting in localized tissue damage. Additionally, IL-1β activates the NF-κB and MAPK signaling pathways, downregulates tight junction protein expression, and consequently increases intestinal permeability (Dai et al., 2025; Kaminsky et al., 2021). Ccl21a primarily regulates immune cell migration and distribution through its receptor CCR7. During inflammatory responses, Ccl21a enhances the severity of inflammation by promoting the accumulation of immune cells at inflammatory sites (Chen et al., 2020; Popa-Fotea et al., 2023). Following the administration of *A. muciniphila* treatment in the *C. albicans* translocated infection group, a notable decrease in IL-1β and Ccl21a levels were recorded, thereby mitigating the inflammatory response. These observations were confirmed in Fig. S2.

In summary, this study developed mouse models for *C. albicans* intestinal colonization and immunosuppressant-induced translocated infection to better replicate human intestinal *C. albicans* infection. Additionally, the research demonstrated that *A. muciniphila* treatment can improve intestinal flora and metabolite composition, augment the tight junctions of colonic tissue, and diminish systemic inflammatory response. This presents a novel therapeutic approach for potential treatment of intestinal *C. albicans* infection using *A. muciniphila*. Nevertheless, the exact mechanism requires further investigation.

## Figures and Tables

**Table 1.** Statistical analysis results of the primary metabolites altered in the colonic contents of mice in the *C. albicans* colonization group compared to the *A. muciniphila* treatment *C. albicans* colonization group (n = 6)

Metabolite	Fold change	*P *value	VIP	Trend
Pyridoxamine 5-phosphate	0.116	1.429E-08	2.442	Down
Gartanin	0.072	7.767E-06	1.773	Down
Matairesinol	7.494	1.815E-05	2.621	Up
3-Ethylphenol	0.180	2.881E-05	1.905	Down
Xylometazoline	3.346	0.0001	1.222	Up
(-)-Epigallocatechin	6.410	0.0001	5.474	Up
N-Acetylhistamine	7.736	0.0001	5.696	Up
N-Acetylneuraminic acid	4.231	0.0002	6.840	Up
7.alpha.-hydroxy-4-cholesten-3-one	2.347	0.0003	2.257	Up
(-)-Secoisolariciresinol	0.171	0.0004	1.217	Down

**Table 2.** Results of statistical analysis of the main metabolites changed in mice intestinal contents for *C. albicans* translocation infection group versus *A. muciniphila* treatment *C. albicans* translocation infection group (n = 6)

Metabolite	Fold change	*P* value	VIP	Trend
Mestranol	0.037	2.341E-10	8.945	Down
Chloroquine	0.232	2.374E-07	1.001	Down
Terbutaline	13.355	2.925E-07	1.109	Up
Prostaglandin i2	28.810	1.638E-06	3.344	Up
Dl-4-hydroxy-3-methoxymandelic acid	0.056	3.854E-06	1.552	Down
Isoproterenol	6.119	5.012E-06	1.189	Up
Matairesinol	0.054	9.886E-06	2.493	Down
6-Hydroxynicotinic acid	8.397	1.510E-05	1.319	Up
Cytosine	5.108	3.050E-05	5.014	Up
Inosine	11.350	3.053E-05	7.661	Up

## Data Availability

The raw data from the 16S rRNA gene sequence and transcriptomic experiment have been deposited in the SRA database of NCBI under the accession numbers PRJNA1093079 and PRJNA1094909 (https://www.ncbi.nlm.nih.gov/guide/). The raw data from the metabolomic experiment can be found at Metabolights under the accession number MTBLS10105 (https://www.ebi.ac.uk/metabolights/editor/login).

## Supplementary Information

Table S1. Primers used for RT-PCR

Fig. S1. Validation of the animal models. (A) Quantitative assessment of *C. albicans* in the intestinal contents of the four experimental mice. (B–C) Augmentation of the 18S gene of *C. albicans* in the colonic tissue and renal organs of the test mice. (D) Fungal immunofluorescence staining of colonic tissue in the Control group, the scale bar is indicated in pixels, with 1,000 pixels corresponding to 1 μm. (E) Fungal immunofluorescence staining of colonic tissue in the *C. albicans* colonized group, the scale bar is indicated in pixels, with 1,000 pixels corresponding to 1 μm. (F) Fungal immunofluorescence staining of the kidney in the group infected with translocated *C. albicans*, the scale bar is indicated in pixels, with 500 pixels corresponding to 1 μm. (G) Fungal immunofluorescence staining of *C. albicans*, the scale bar is indicated in pixels, with 1,000 pixels corresponding to 1 μm (^*^*P* < 0.05, ^**^*P* < 0.01).

Fig. S2. The AKK intervention modifies the expression of intestinal barriers, immune cells, and inflammatory factors in mice. (A) Staining of mouse colonic tissue in each experimental group for HE, CD68, MPO, CD56, and CD19. (B–D) Statistical analysis of inflammatory factors and closure proteins in mice across all experimental groups. (E) Statistical analysis of immune cell expression in the colonic tissue of mice from each experimental group (^**^*P* < 0.01).
