# Supplementary material for: Multi-omics to evaluate the protective mechanisms during Akkermansia muciniphila treatment of Candida albicans colonization and subsequent infection
Source: J Microbiol. 2025 Aug 31;63(8):e2502007. doi: 10.71150/jm.2502007 (PMC13577117; doi:10.71150/jm.2502007)
Supplement: Supplementary file 1 [file jm-2502007-Supplementary-Fig-S1.pdf]

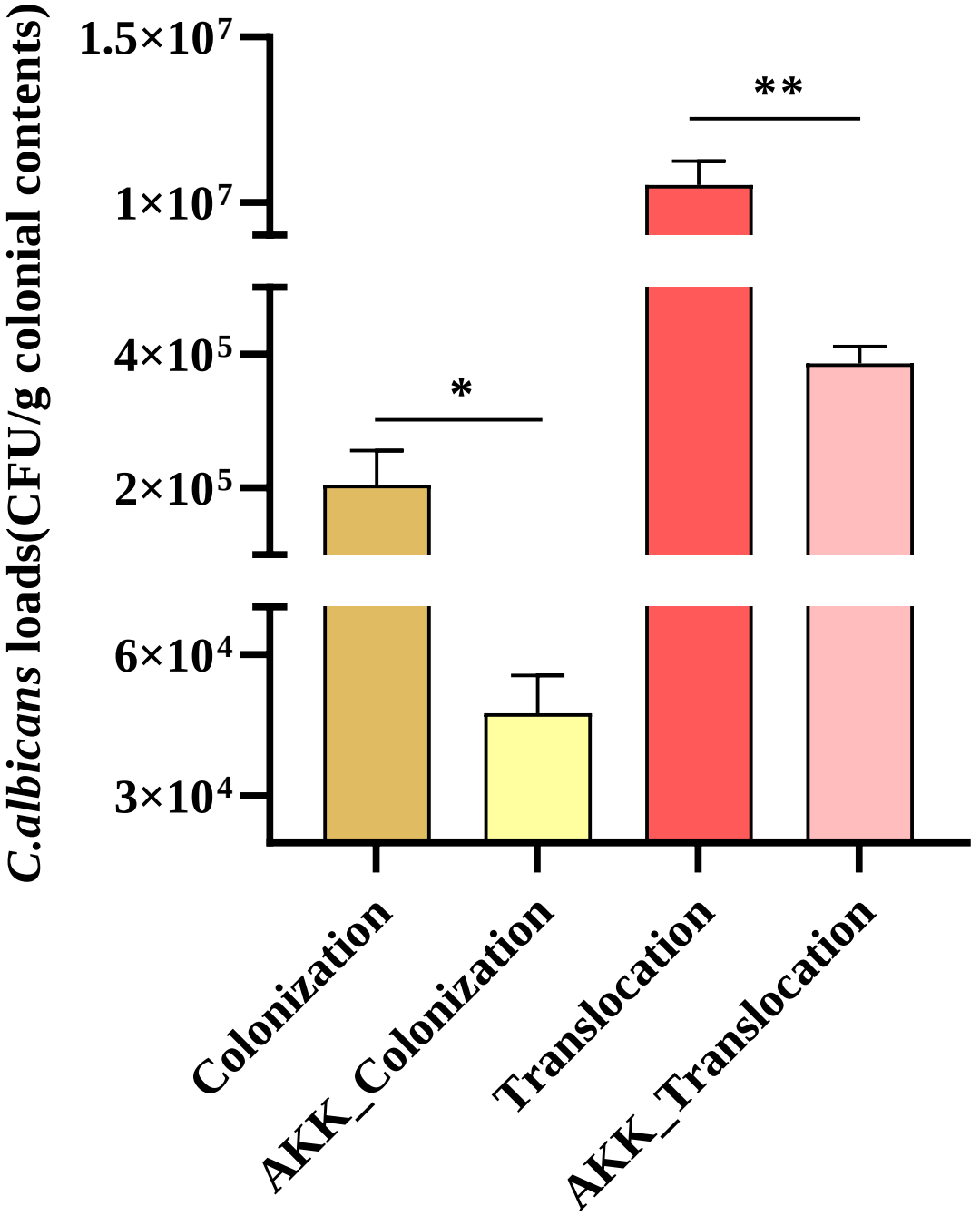

C.albicans loads(CFU/g colonial contents)
1.5×10^7
1×10^7
4×10^5
2×10^5
6×10^4
3×10^4
**
*
Colonization
AKK_Colonization
Translocation
AKK_Translocation

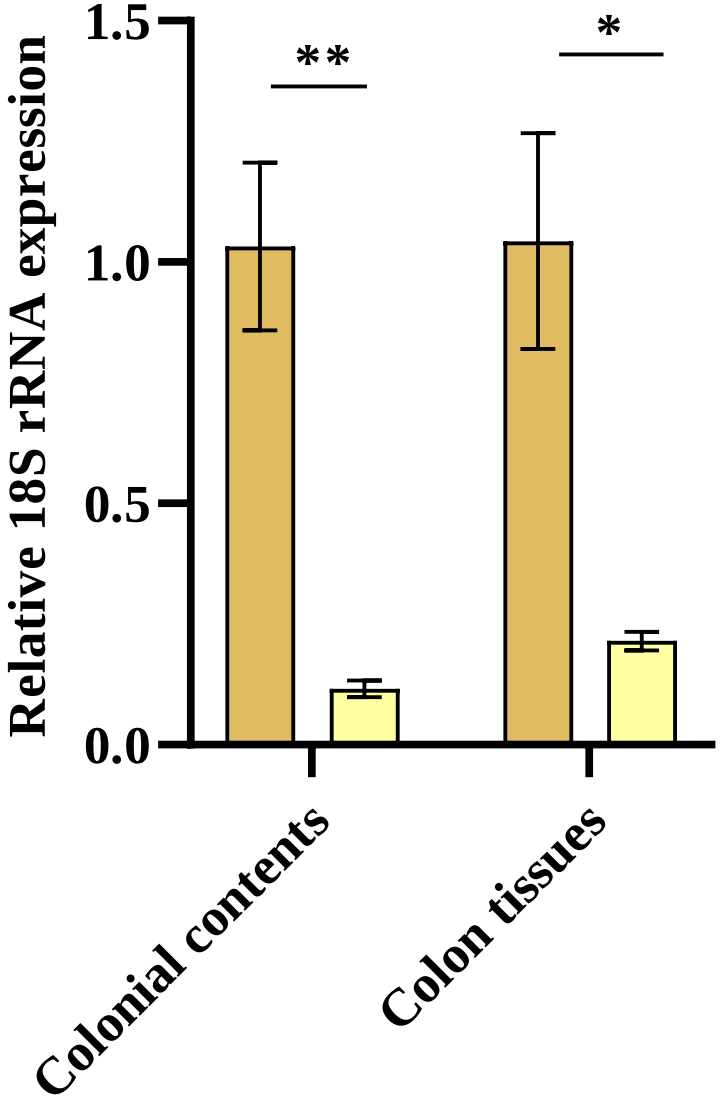

Relative 18S rRNA expression
1.5
1.0
0.5
0.0
**
*
Colonial contents
Colon tissues

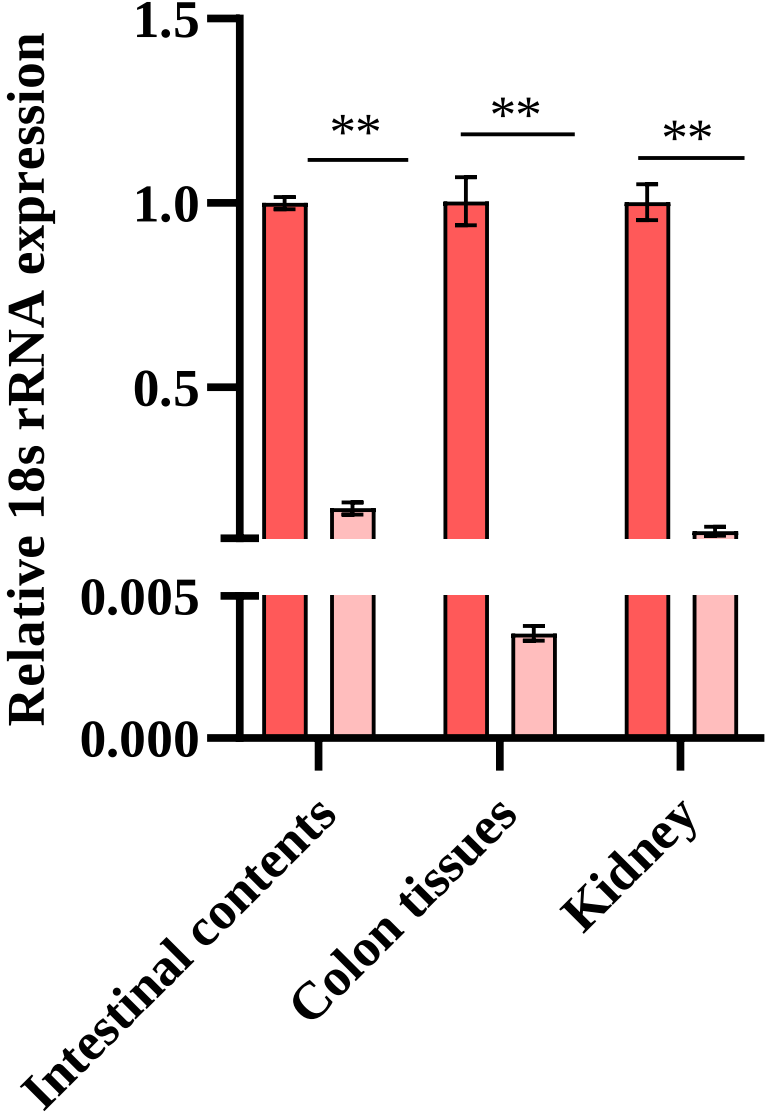

Relative 18s rRNA expression
1.5
1.0
0.5
0.005
0.000
**
**
**
Intestinal contents
Colon tissues
Kidney

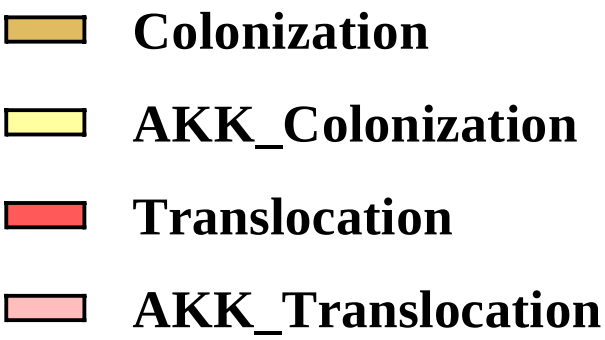
Colonization
AKK_Colonization
Translocation
AKK_Translocation

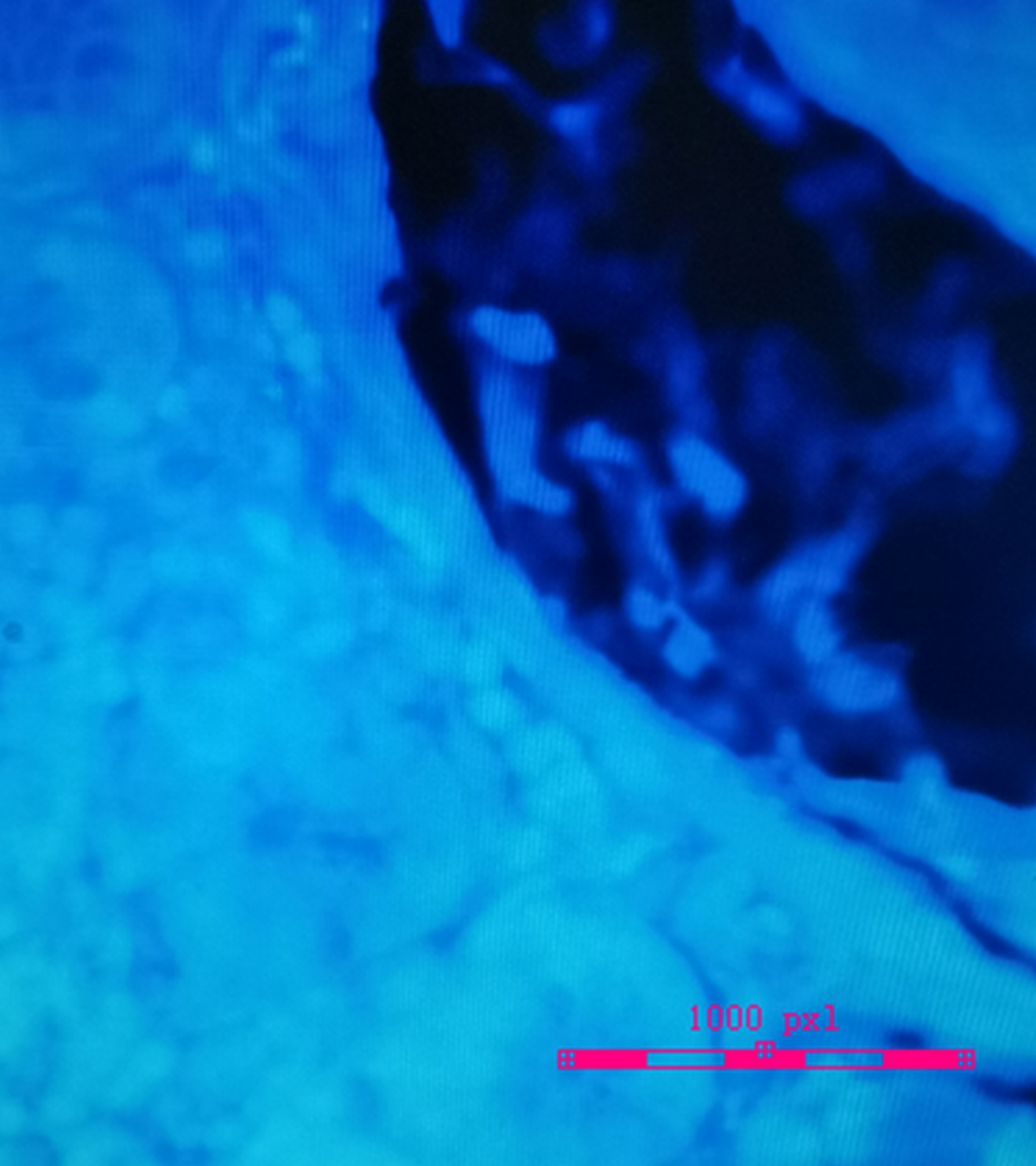
1000 pxl

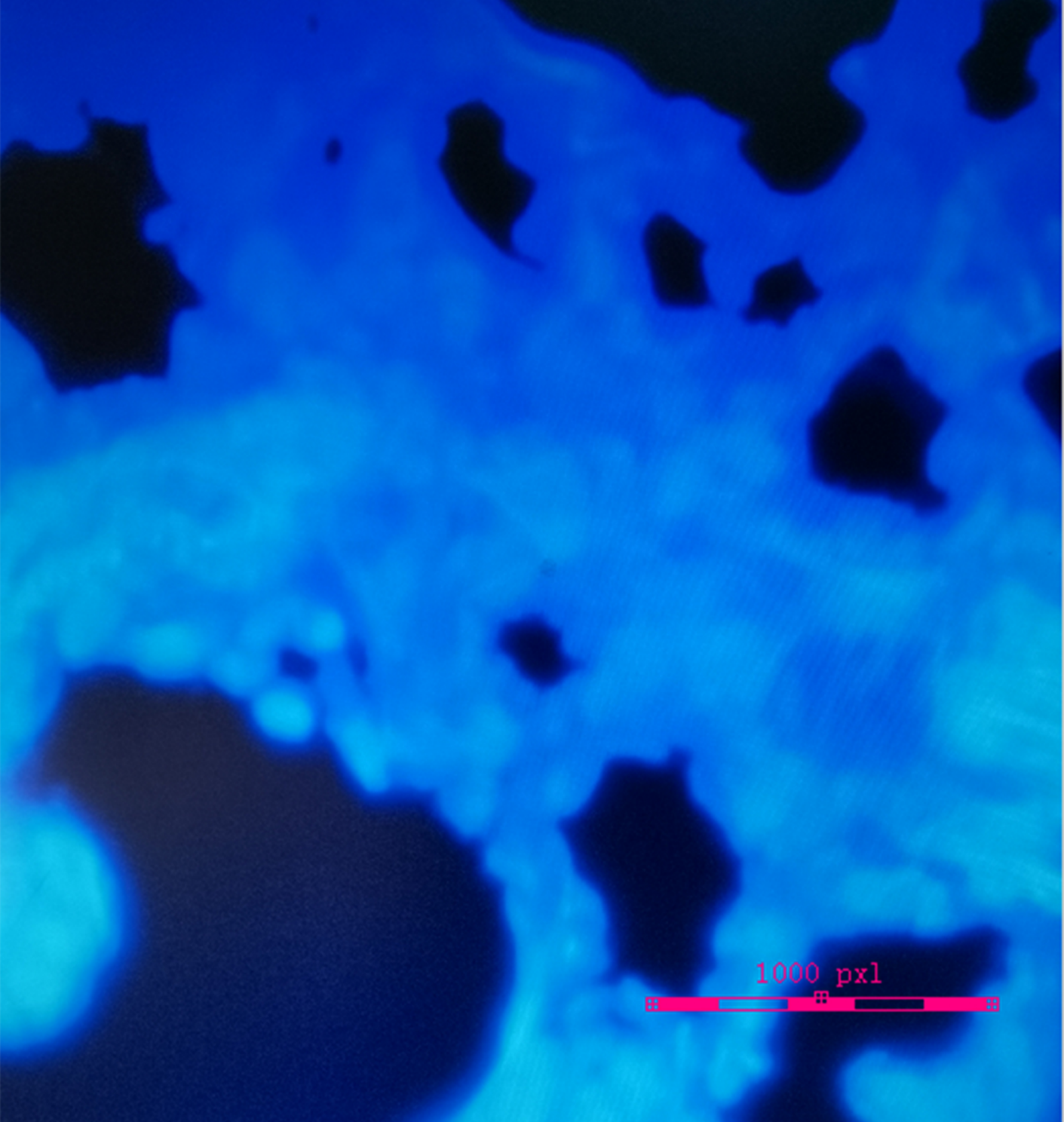
1000 pxl

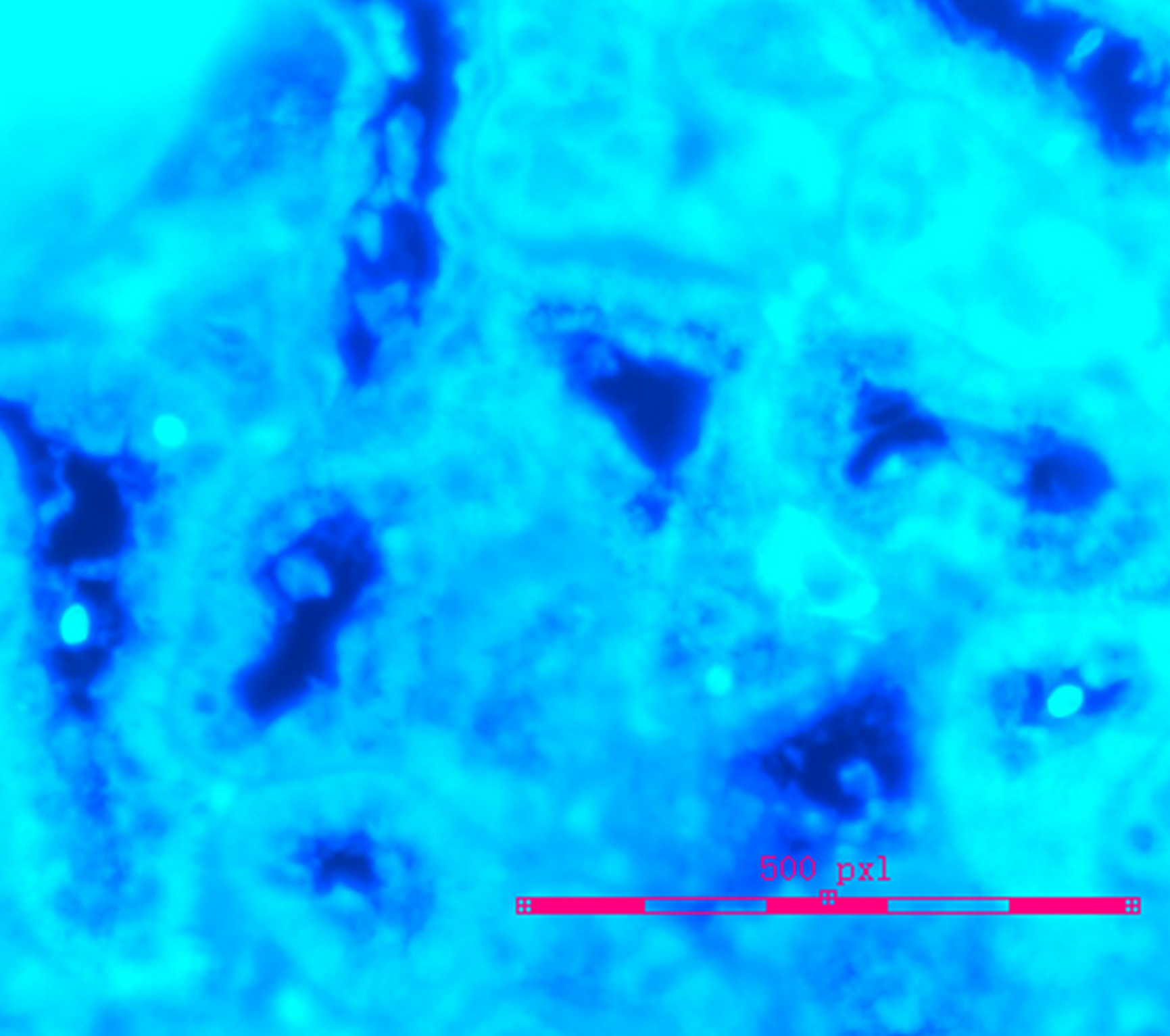
500 pxl

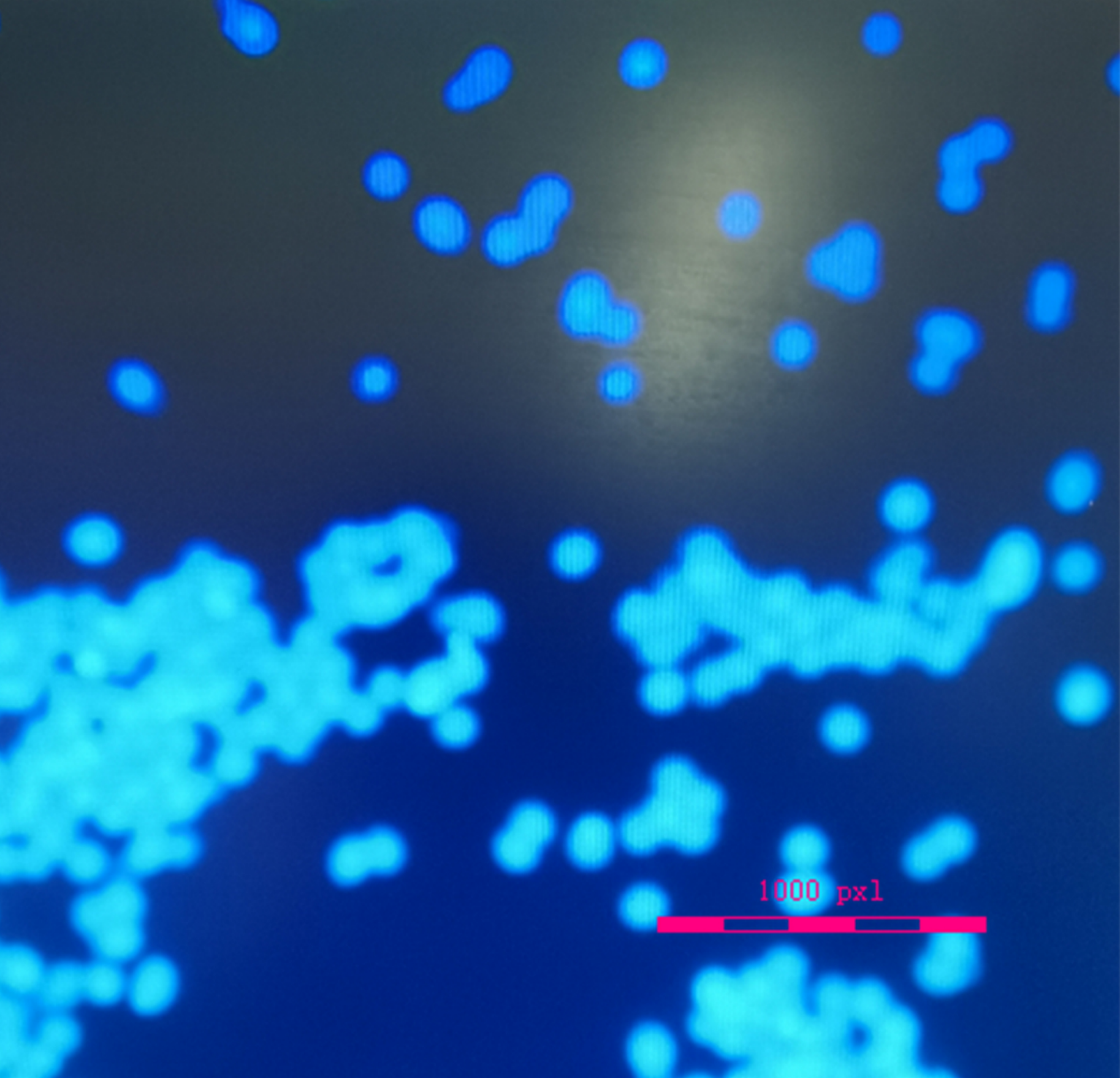
1000 pxl
