# Supplementary material for: Multi-omics to evaluate the protective mechanisms during Akkermansia muciniphila treatment of Candida albicans colonization and subsequent infection
Source: J Microbiol. 2025 Aug 31;63(8):e2502007. doi: 10.71150/jm.2502007 (PMC13577117; doi:10.71150/jm.2502007)
Supplement: Supplementary file 2 [file jm-2502007-Supplementary-Fig-S2.pdf]

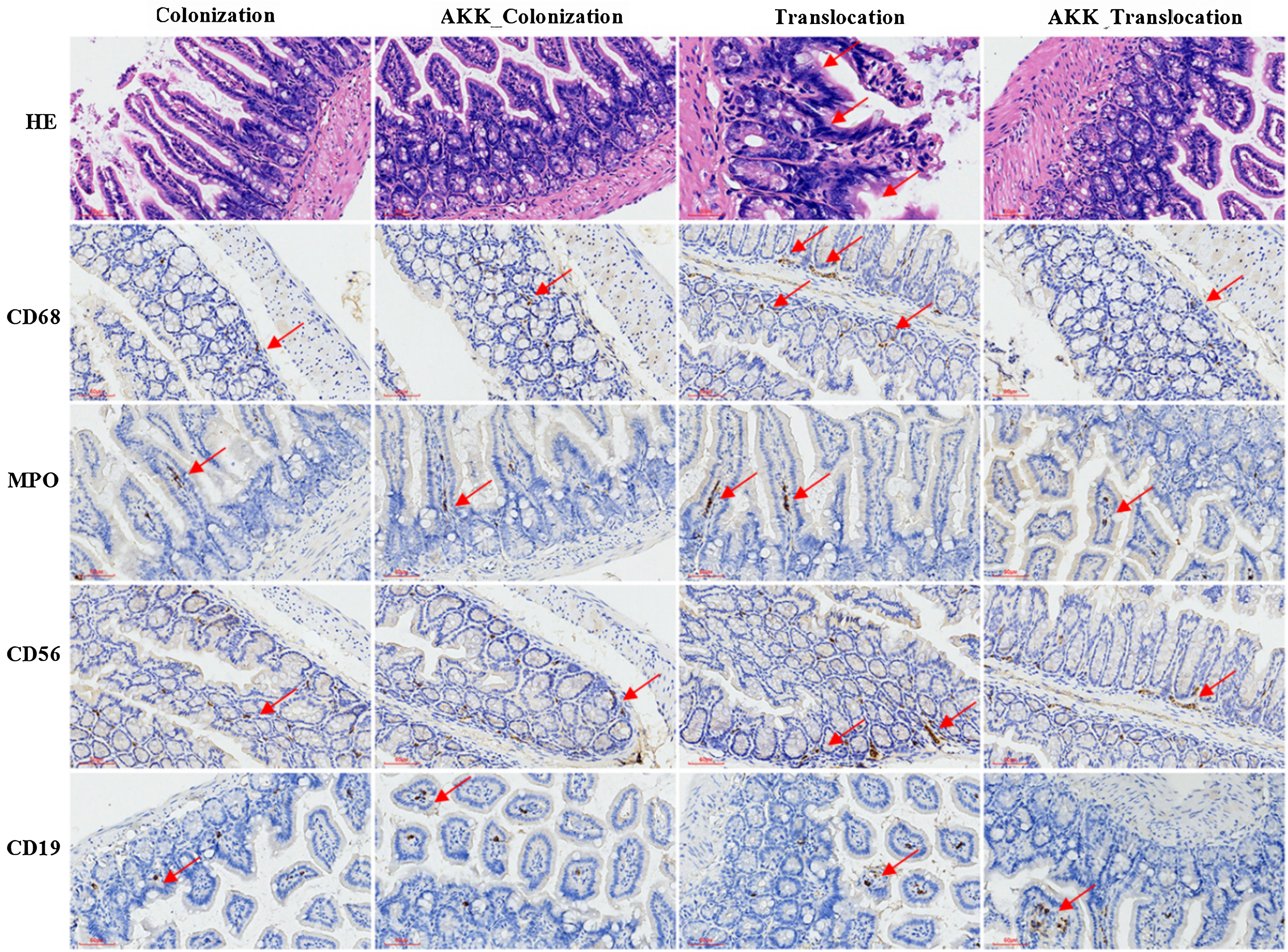
Colonization
AKK_Colonization
Translocation
AKK_Translocation
HE
CD68
MPO
CD56
CD19

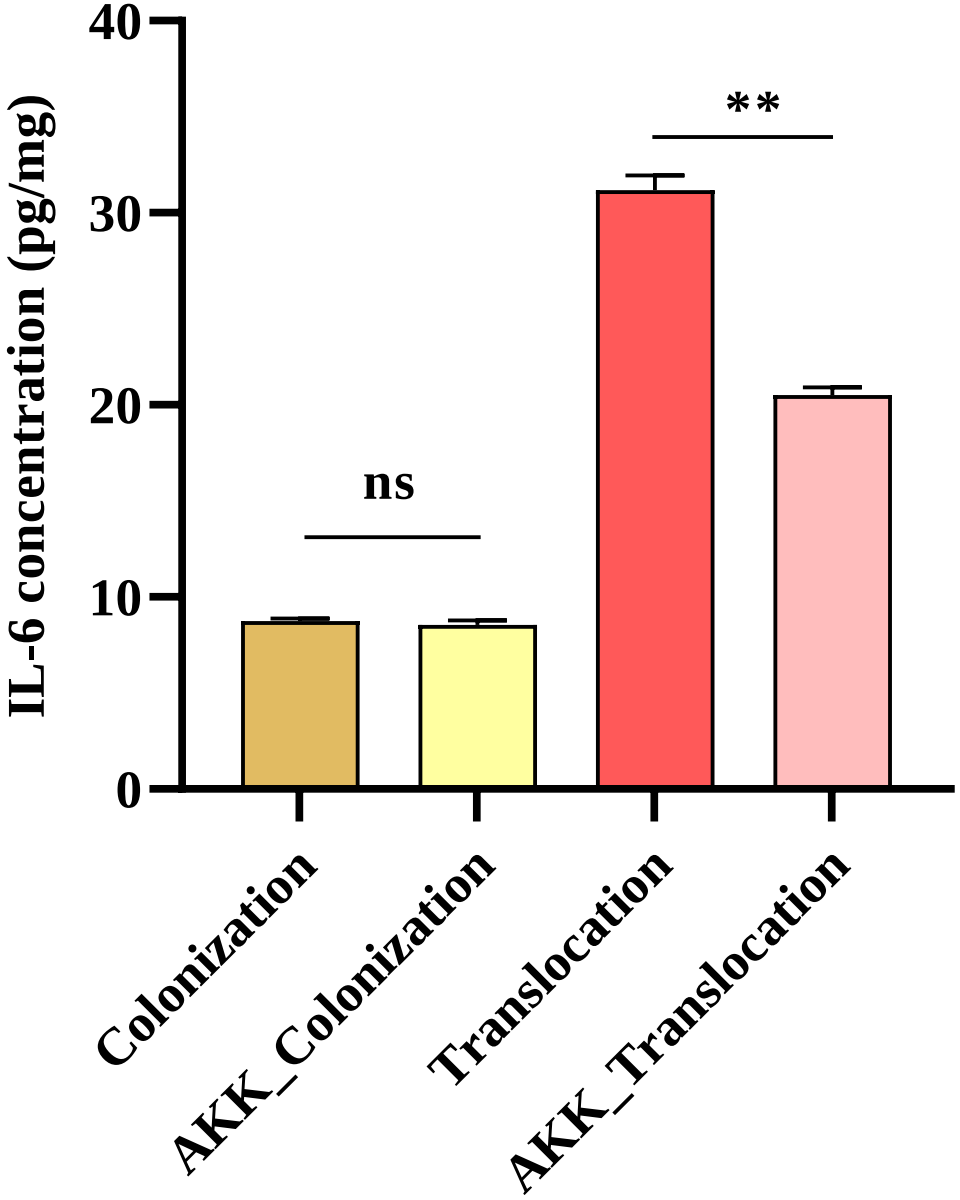

IL-6 concentration (pg/mg)
40
30
20
10
0
ns
**
Colonization
AKK_Colonization
Translocation
AKK_Translocation

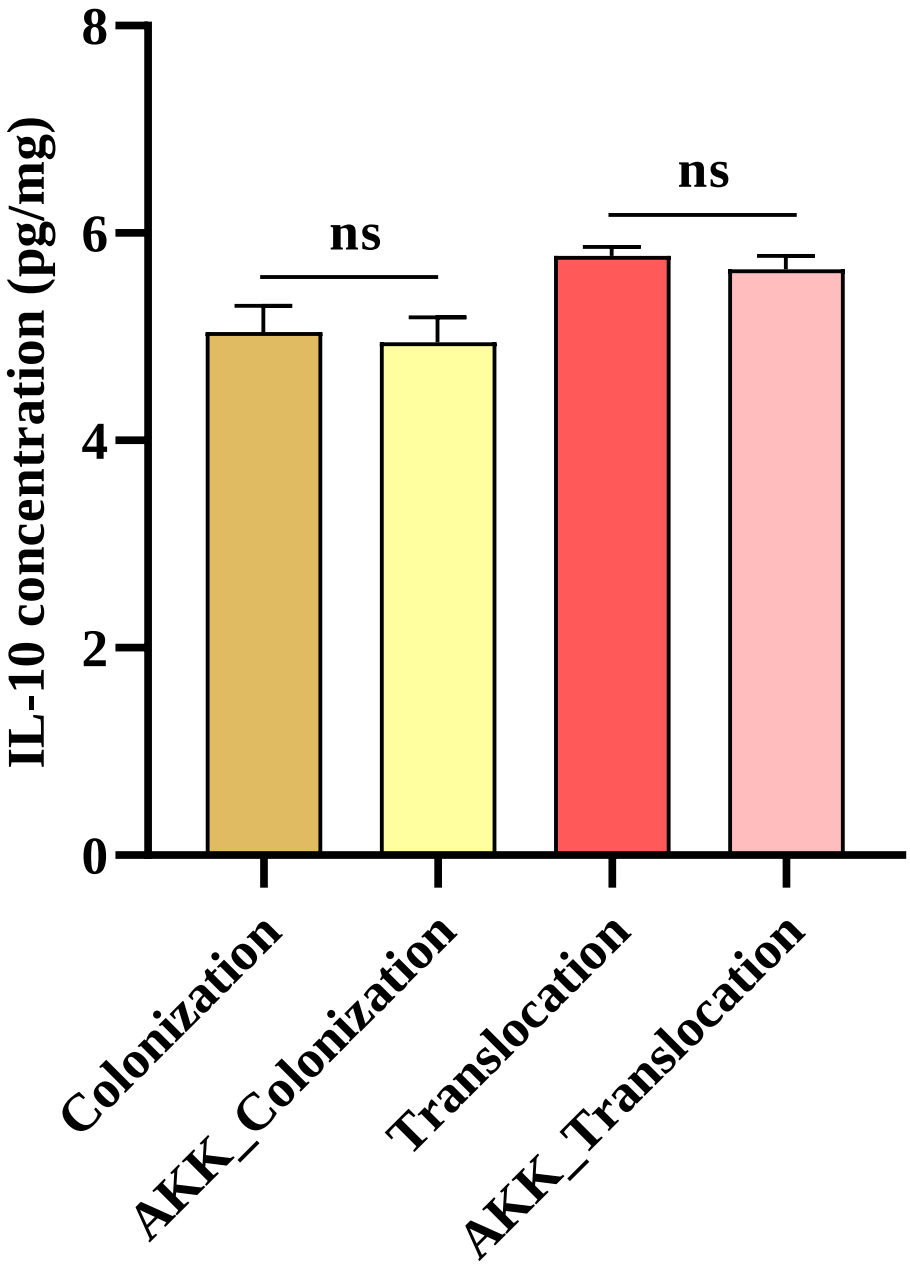

IL-10 concentration (pg/mg)
8
6
4
2
0
ns
ns
Colonization
AKK_Colonization
Translocation
AKK_Translocation

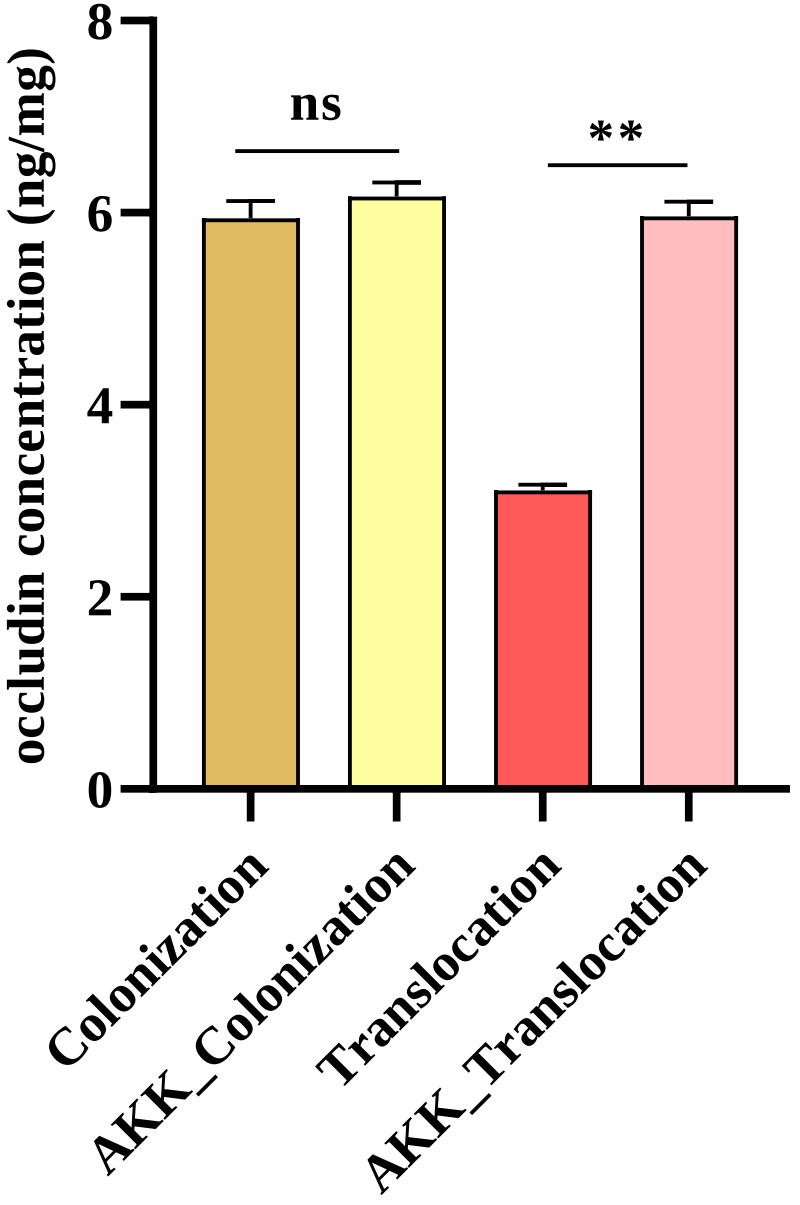

occludin concentration (ng/mg)
8
6
4
2
0
ns
**
Colonization
AKK_Colonization
Translocation
AKK_Translocation

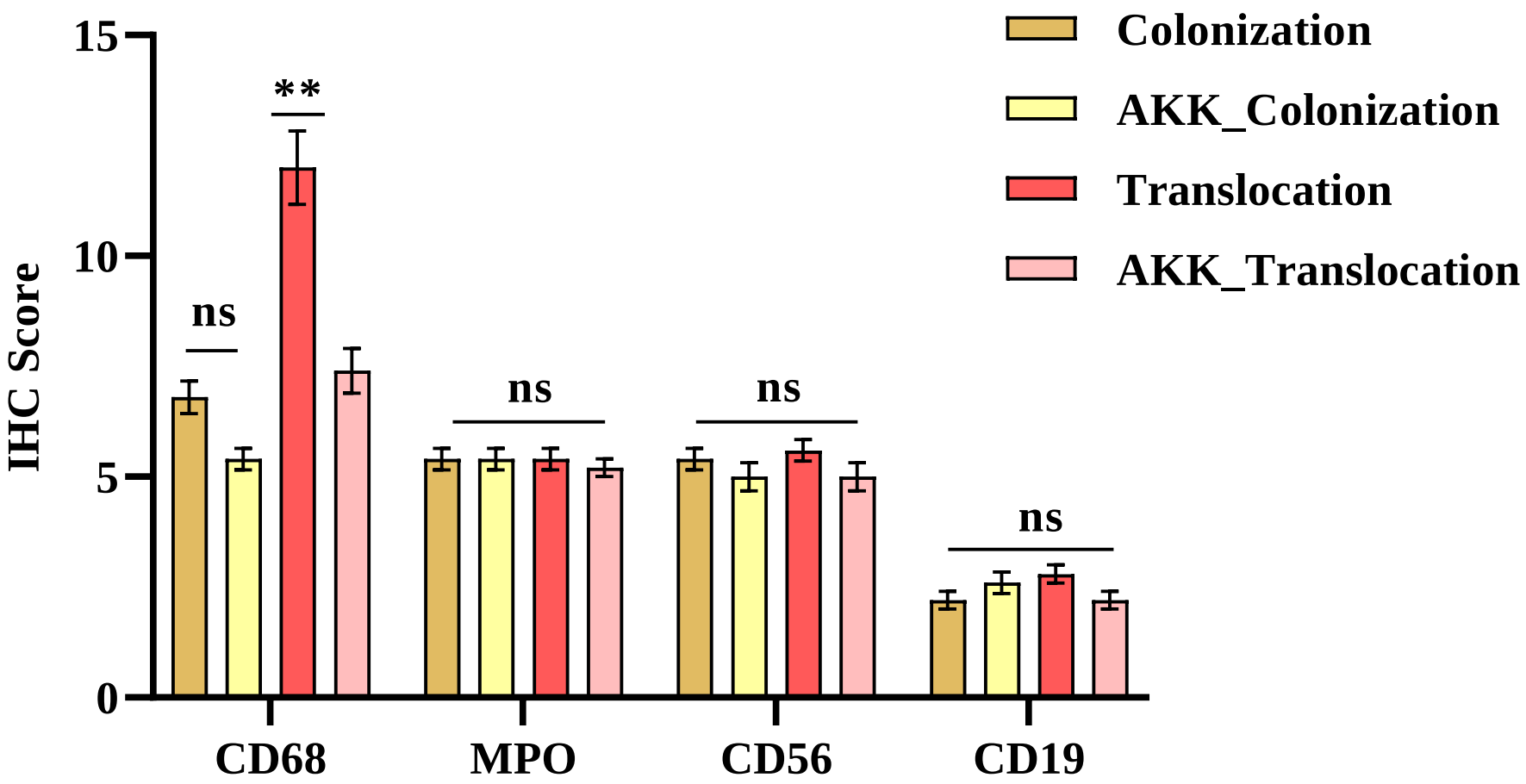
Colonization
AKK_Colonization
Translocation
AKK_Translocation
IHC Score
15
10
5
0
**
ns
ns
ns
ns
CD68
MPO
CD56
CD19
