# Supplementary material for: Multi-omics to evaluate the protective mechanisms during Akkermansia muciniphila treatment of Candida albicans colonization and subsequent infection
Source: J Microbiol. 2025 Aug 31;63(8):e2502007. doi: 10.71150/jm.2502007 (PMC13577117; doi:10.71150/jm.2502007)
Supplement: Supplementary file 3 [file jm-2502007-Supplementary-Table-S1.pdf]

**Table S1.** Primers used for RT-PCR

| Gene symbol | Forward primer (5'-3') | Reverse primer (5'-3') |
|---|---|---|
| GAPDH | CGTGCCGCCTGGAGAAACCTG | AGAGTGGGAGTTGCTGTTGAAGTCG |
| 18S | GAGGTCTAAACTTACAACC | CGTTGTTGAAAGTTTTGAC |
| IL-1β | GCAACTGTTCCTGAACTCAACT | ATCTTTTGGGGTCCGTCAACT |
| Ccl21a | AACTCAACCACAATCATG | CTTGAAGTTCGTGGGGGA |
| Plau | TGCCCACAGACCTGATGCTATTA | GCATTCTTGGACAAACTGCCTTA |
